# Spatial microscopic modeling of collective movements in multi-robot systems: Design choices and calibration

**DOI:** 10.3389/frobt.2022.961053

**Published:** 2022-10-06

**Authors:** Cyrill Baumann, Alcherio Martinoli

**Affiliations:** Distributed Intelligent Systems and Algorithms Laboratory, School of Architecture, Civil and Environmental Engineering, École Polytechnique Fédérale de Lausanne (EPFL), Lausanne, Switzerland

**Keywords:** modeling, flocking algorithm, system identification (SI), collective movements, multi-robot system (MRS)

## Abstract

Despite the strong increase in available computational power enabling an unprecedented level of realism in simulation, modeling robotic systems at higher abstraction level remains crucial to efficiently design robot controllers and analyze their properties. This is especially true for multi-robot systems, with their high computational complexity due to the numerous interactions among individual robots. While multiple contributions in the literature have proposed approaches leading to highly abstracted and therefore computationally efficient models, often such abstractions have been obtained with strong assumptions on the underlying spatiality of the system behavior (e.g., well-mixed system, diffusive system). In this work, we address the modeling of an arbitrary collective movement involving the displacement of a robot ensemble along a certain trajectory overlapped with continuous interactions among the robotic members. Without loss of generality, we have focused our modeling effort on a flocking case study, as a prominent and well-known example of collective movement. We investigate our case study at the microscopic level while leveraging a more faithful submicroscopic model (implemented through a high-fidelity robotic simulator) as ground-truth. More specifically, we illustrate multiple choices for designing and calibrating such microscopic models, so that their faithfulness with the underlying submicroscopic model of the same physical system is preserved. Such effort has produced concrete implementations of three different microscopic models for the same case study, all taking into account the spatiality of the collective movement. We find that all three microscopic models produce quantitatively accurate estimations for our flocking case study. As they rely on different underlying assumptions and modeling techniques, the choice between them is a trade-off between the computational cost, the modeling effort, the metrics considered to evaluate their faithfulness, and the subsequent usage (e.g., control design, system property analysis, control code prototyping).

## 1 Introduction

As defined by Hamann (2010) in the scope of swarm robotics, a model of a swarm of robots is an “as far as possible mathematically closed form that predicts the behavior of large self-organizing robot groups based on their control algorithm.” While also concerned with smaller groups of robots, this definition remains valid for Multi-Robot Systems (MRSs). In fact, such modeling is crucial not only for efficiently designing robot controllers, but also to analyze and predict the behavior of an MRS for a given controller. Therefore, the robot control algorithm should be exposed to such models, that is, either being used as input for the model or being directly integrated into the model structure.

This work is concerned with modeling of collective movements; more specifically, our objective is, without loss of generality, to accurately model a flocking case study. Therefore, we will focus the remaining of this introduction on presenting the different types of model typically used for MRSs and swarm robotics and point out which techniques have been used for modeling flocking in previous works.

### 1.1 Modeling levels and techniques for multi-robot systems

Computational efficiency is one of the main motivations for the use of models of higher abstraction over realistic simulations or even real robot experiments. Consequently, the most commonly used modeling technique are mean-field macroscopic models, due to their ability to capture the ensemble of the MRS independently of the number of robots involved. Lerman et al. (2005) and more recently Elamvazhuthi and Berman (2019) provide a thorough overview of the wide range of different mean-field macroscopic modeling techniques used in the scope of swarm robotics. Although these methods are computationally efficient, they have often been designed in the context of modeling fluidic, molecular, or purely mechanical systems and, therefore, rarely expose explicitly the underlying control structure and related parameters inherent to robotics. This in turn significantly reduces the effectiveness of leveraging such modeling methods to solve a crucial inverse engineering problem in MRS: given the overall collective behavior and possibly related quantitative performance metrics, design individual robotic nodes so that the desired system behavior is produced and the resulting performance optimized. Three additional difficulties make such inverse engineering problem hard: first, starting from macroscopic models and going down to physical reality (possibly not only designing or optimizing software but also hardware features) means also bridging a large simulation-to-reality gap; second, the nodes of an MRS are typically affected by severe resource constraints (this is particularly true for most of the swarm robotics demonstrators), and it is therefore rarely possible, for instance, to exploit canonical layering techniques outsourcing the implementation of high-level desired node behavior to well-separated low-level control layers; third, most of the macroscopic modeling techniques borrowed from other fields (e.g., statistical physics, molecular chemistry) target underlying continuous systems while the reality of MRS nodes is typically hybrid, namely involving continuous and discrete components Henzinger (1996).

Promising attempts to tackle these difficulties are represented by multi-level modeling methods, such as Egerstedt et al. (1999), Martinoli et al. (2004) or Brambilla et al. (2014). In Martinoli et al. (2004), the authors use Probabilistic Finite State Machines (PFSMs) with transition probabilities calibrated on physical experiments in order to faithfully model an MRS using the control FSM as model state-space. They also introduce a multi-level hierarchical representation of the modeling technique, which we adopt for this work: submicroscopic models (high-fidelity simulation of reality), microscopic models (each agent is individually represented, but only relevant robot features are captured), and macroscopic models (representation of the whole MRS, typically corresponding to a mathematical model of the dynamical system representing the group). In their framework, called Multi-Level Modeling (MLM), model structure and parameters are generated manually in a bottom-up fashion, from the physical reality to the highest abstraction level (i.e., the macroscopic representation) leveraging the FSM-based controller blueprint for the structure and dedicated experiments for the parameter calibration.

In Egerstedt et al. (1999), a microscopic model consisting of regularized hybrid automata is proposed. Similarly to the work in Martinoli et al. (2004), it is obtained in a bottom-up fashion, basing its structure on the underlying robotic controller. This work is then extended to hierarchical hybrid automata with formal model checking in Furbach et al. (2008) and improved in Mohammed and Furbach (2010) to reduce the size of the model by using constraint logic programming. The overall framework (conveniently summarized in Mohammed et al. (2010)) uses parallel hybrid automata to represent individual parts of the system (e.g., robots, environment, etc.) which communicate through shared variables or synchronized transitions. Together they form a composed, hierarchical automata representing the whole system (thus arguably corresponding to a macroscopic view), which can be processed through model checking techniques to investigate, for example, the reachability of a certain environmental state.

In Brambilla et al. (2014), a property-driven design method is proposed for the manual top-down design of robotic controllers. Starting from desired (macroscopic) properties of a system, a macroscopic model of the MRS is derived iteratively. In a second step, a microscopic model is derived from the macroscopic model using again an iterative process. While, in principle, the method is agnostic to the used modeling techniques for both macroscopic and microscopic levels, the authors demonstrate the design method using deterministic Markov chains and probabilistic computation tree logic, resulting thus in similar models as the previous frameworks. This similarity in the resulting models seems natural, given that leveraging (probabilistic) FSMs switching between continuous-time control algorithms is a highly intuitive and widely used method for controlling robots, including those belonging to an MRS. This method is formally known as hybrid automata and was first proposed in Henzinger (1996). Naturally, this suggests that one also uses the states of the control FSMs as state space for modeling purposes, which leads to these promising modeling frameworks for MRSs.

In subsequent works, different variations of the frameworks have been explored, such as the use of Chemical Reaction Networks (CRNs) in Matthey et al. (2009), which are then combined with trajectory matching in Mermoud et al. (2012) to automatically generate a macroscopic model without knowledge of the implemented control architecture. However, this implies the need for the observability of the trajectories of the individual robotic nodes to build the interaction graph. Additionally, it does not preserve any strict mapping with the underlying physical and technological constraints, which limits the use of the resulting model for control design. Haghighat et al. (2019) leverages the same trajectory matching technique, but uses it to automatically calibrate parameters of a stochastic submicroscopic model of floating robots, while using a microscopic model for designing the ruleset eventually deployable on both high-fidelity simulation and real robots. In Hamann et al. (2014) the authors establish a relationship between a microscopic model consisting of CRNs and a macroscopic model using stochastic differential equations, which is applied to a collective decision-making process of a swarm of agents. In Reina et al. (2015) high-level guidelines are provided for the inverse problem: designing a microscopic model, given the desired macroscopic dynamics. While the considered case study of the shortest path selection is spatial, all spatiality needs to be neutralized through dedicated tweaking of the individual robots’ behavior in order to obtain the desired quantitative micro-macro link.

Another modeling technique, borrowed from biochemical systems, is proposed in Ciocchetta and Hillston (2009). In this framework, the system is only modeled at the microscopic level. Originally created for the analysis of biochemical systems, it is based on stochastic simulation, fluid flow analysis, and statistical model checking, which allows it to verify specific macroscopic characteristics. It has been applied to robotic swarms in Massink et al. (2013), for example. However, it shares the same limitations as CRNs as no strict mapping with the underlying physical and technological parameters, respectively constraints, is preserved.

Petri nets are somewhat similar to a combined view of all FSMs of the MLM framework at a microscopic level. Similarly to FSMs, they are event-based deterministic structures: a transition to another state occurs as soon as all input states are occupied. Thus, a Petri net can reproduce the behavior of a FSM by integrating the environment into the net, such that the required environmental conditions for FSM-state-transitions are input states for the state transitions within the Petri net. In Costelha and Lima (2008) they have been used to analyze the behavior of an MRS playing robotic soccer. In Pereira et al. (2014), generalized stochastic Petri nets are used in combination with mean-field dynamics analysis to model on a macroscopic level an MRS controlled by institutional controllers based on executable Petri nets, that is, Petri nets taking into account robot actions and sensing.

Another noteworthy category of modeling techniques used frequently for MRS are high-fidelity and multi-agent simulators whose capabilities, such as preservation of spatiality, will be discussed in the following section.

### 1.2 Preserving spatiality in models

A major assumption common to all models presented in the previous section is the well-mixedness of the system. Well-mixedness of a SRS can be translated into the presence of a large number of stochastic interactions in the scenario, which is often not the case in collective movements, as the spatial distribution of the agents is actively controlled and fairly deterministically. One possible solution to this problem is presented in Correll and Martinoli (2006), where system identification techniques are used to compensate, through optimization of the model parameters, for the deviation from the well-mixedness assumption used to design the initial model structure.

Another possibility of preserving spatiality at the macroscopic level consists in the use of the Fokker-Planck equation. This technique provides a probability density for a particle, or robot in our case, to be at a given position at a specific time. This has been applied successfully to MRSs in Hamann and Wörn (2008) and Prorok et al. (2011), though, to our knowledge, it has never been applied to flocking. In Elamvazhuthi et al. (2018) reaction-advection-diffusion equations are used to optimize robotic control parameters for an MRS coverage scenario. Unfortunately, a major drawback of such techniques based on partial differential equations is their dependence on a high degree of stochasticity within the system, which is not the case for our case study. Furthermore, the underlying robotic control structure and related parameters are mostly abstracted, complicating the use of such models for control design, though their applicability for parameter optimization has been demonstrated on multiple occasions.

In summary, while most of the works mentioned so far maintain a degree of spatiality in their models, to the best of our knowledge, none exists that maintains enough spatiality to model spatially coordinated groups of robots, such as our chosen flocking case study.

A different category of tools, dynamic robot simulators, usually maintain full spatiality and model both robots and their environment more or less closely to reality. A survey of dynamic simulators based on user feedback can be found in Ivaldi et al. (2014). In this work, we distinguish between two types of simulators:

• High-fidelity simulators, leveraged for implementing submicroscopic models (fat), aim at emulating reality as closely as possible, usually using dedicated physics engines. Popular examples are Webots Michel (2004), Gazebo Koenig and Howard (2004) or ARGoS Pinciroli et al. (2012). While highly reliable, these models unfortunately only provide a limited speedup in comparison to reality (1-2 orders of magnitude) and are thus ill-suited for analyzing and predicting the behavior of MRSs consisting of large numbers of robots.
• Spatial microscopic simulators, leveraged for implementing absolute microscopic models (fat), are often custom implementations using Matlab. They simulate the robots as point masses with kinematics or even as fully holonomic particles. Although faster than sub-*μ*M, these models usually broadly simplify physics (e.g., no friction), which, depending on the scenario, leads to significant reality gaps, that is, differences between the simulation and reality. Furthermore, the speedup achievable with *μ*M-A models also decreases significantly for higher numbers of robots.

### 1.3 Modeling collective movements

The original flocking work of Reynolds (1987), but also Vicsek et al. (1995), both use kinematic *μ*M-A models to demonstrate their algorithms. However, also more recent works, such as Olfati-Saber (2006), Vásárhelyi et al. (2018), Micklisch et al. (2018), or Jia and Vicsek (2019) rely on μM-A models to demonstrate their flocking controller’s performances. It should be noted that the definition of “flocking model” does not correspond to the definition of models used in this work. Indeed, their “flocking model” corresponds to our “flocking controller”. Works that implement flocking on real robots, such as Hayes (2002) or Navarro et al. (2008), tend to use submicroscopic simulators instead, presumably in order to reduce the simulation-to-reality gap. However, there are contrasting examples, such as Virágh et al. (2013) or Shirazi and Jin (2017) where custom *μ*M-A simulators are used.

As motivated expertly in Hamann (2018), simulating spatial displacements is computationally expensive even when using the less precise *μ*M-A, particularly for a large number of agents. By simplifying the interactions between agents with viscoelastic models, that is, virtual, “mechanical” links composed of springs and damping elements, techniques borrowed from physics can be applied. Using a fractional-order model (Heymans and Bauwens (1994)), significant computational speedups have been achieved in Goodwine (2014) and subsequent works. However, while such models have been successfully applied to detect faulty robots in Ni and Goodwine (2020), their applicability for robot control design or analysis remains unknown.

Another set of works leverages modeling for the analysis of MRSs, usually focusing on control or stability analysis. In this case, the MRS is modeled for the target purpose, and therefore all other aspects of the system such as embodiment, noise, etc., are removed. Often, Lyapunov theory is used to prove stability, such as in Gazi and Passino (2005) or Schwager et al. (2011). While extreme computational cost reductions are achieved, the underlying control structure and related parameters are completely abstracted, making the use of these models for other purposes such as control design or optimization difficult.

### 1.4 Contributions

As mentioned previously, a major gap in the current literature of MRS modeling is related to computationally efficient representations of tightly coordinated systems in space, such as collective movements. Indeed, to our knowledge, no microscopic (and consequentially no macroscopic) model exists capable of maintaining the spatiality needed for this purpose while capturing the stochasticity of interactions typical of a real MRS in a computationally efficient way. As reported above, currently to this purpose essentially only microscopic and submicroscopic simulators tracking positional states in global coordinates have been adopted. However, these modeling instruments are computationally expensive due to their rich and continuous tracking of system states and, therefore, do not scale well with the size of the MRS. Moreover, such modeling instruments are not designed with further abstraction levels in mind, and it is therefore difficult to formalize microscopic-to-macroscopic links starting from their rich representations. As a consequence, while a macroscopic model is computationally cheaper and more scalable than a microscopic model, and thus often the desired final product of a modeling effort, microscopic modeling choices play a crucial role in the overall abstraction process, as they serve as the link between macroscopic and submicroscopic representations, or put it differently, between mathematical formalism and physical reality of an MRS. However, despite the highly influential role of such choices in the whole modeling process, we believe that the microscopic modeling level has not received sufficient attention to date from the research community in MRSs. This motivated us to focus this contribution precisely on such modeling level and investigate various options to produce valuable representations for its crucial linking function.

The main contributions of this work are two-fold. First, we demonstrate how to apply a canonical microscopic model ***μ*** **M-C** to a flocking case study using a discretization of the state space. Combining geometrical reasoning similar to Winfield et al. (2008) and calibration using system identification techniques as in Correll and Martinoli (2006), we then calibrate the model to compensate for the non-well-mixedness of the system. Second, we introduce a novel microscopic model ***μ*** **M-R**, situated between *μ*M-A and *μ*M-C, which is capable of modeling the same case study without introducing a discretization of the state space. We compare both resulting microscopic models against the next-most accurate model, *μ*M-A, commonly used for modeling flocking, as well as a more realistic submicroscopic model sub-*μ*M.

As we illustrate explicitly how each model is built, in addition to discuss their respective advantages and drawbacks, we further hope that this work will simplify the reader’s choice and implementation of a suitable model for case studies involving MRSs engaged in tightly coordinated movements in space.

The remainder of this paper is structured as follows: we start by introducing the flocking scenario and the two different metrics used throughout the paper. We then introduce the three microscopic models, demonstrate how they can be applied to the scenario, and report their performance. Finally, we compare and discuss the different modeling techniques before ending with some conclusive remarks.

## 2 Scenario

We consider a simple flocking scenario using pairwise potential functions between *N* homogeneous robots, based on the control law proposed by Olfati-Saber (2006). The flock of robots is further tasked to follow an 8-shaped trajectory, given as a temporal trajectory in a common reference frame. This trajectory can be considered serving an equivalent function of that of the migratory urge introduced in Reynolds (1987).

**FIGURE 1:**
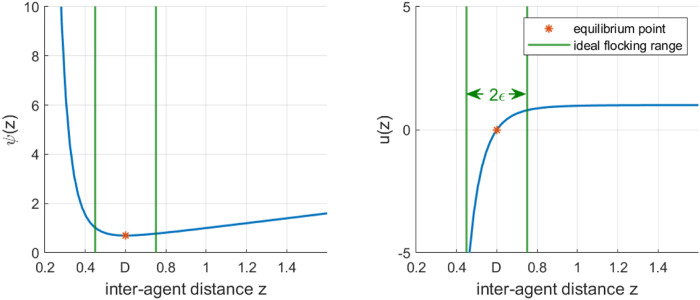
Potential (left) and action function (right) used in this work, with a desired inter-robot distance D=0.6 m. The zone between the green lines is considered the “ideal range” (width of 2*ϵ*) considered in the spatial discretization described in Section 4.

The pairwise potential function is given by Eq. 1 and illustrated in Figure 1, with *z* the distance between the two agents.

$$\psi \left(z\right)=\frac{D^{7}}{6z^{6}}+z$$ (1)

resulting in a pairwise distance control law *u*(*z*) of

$$u\left(z\right)=\frac{d\psi \left(z\right)}{dz}=1-\frac{D^{7}}{z^{7}}$$ (2)

It is trivial to see that Eq. 2 has its equilibrium point at z=D , the desired inter-agent distance within the flock. In this work, we use D=0.6m , which corresponds roughly to four robot diameters in our case (see below for more details on our robots).

Following the implementation framework proposed in our previous work Baumann and Martinoli (2021), we can define $X_{i}^{\mathrm{tar}}=\frac{1}{N\text{\_}ngb}\sum u\left(z_{j}\right)$ the target position of robot *R*_*i*_ within the flock, with *N*_*ngb* being the total number of neighbors of robots *R*_*i*_. For further implementation details, such as the concrete robot control law, we refer to our previous work.

**FIGURE 2:**
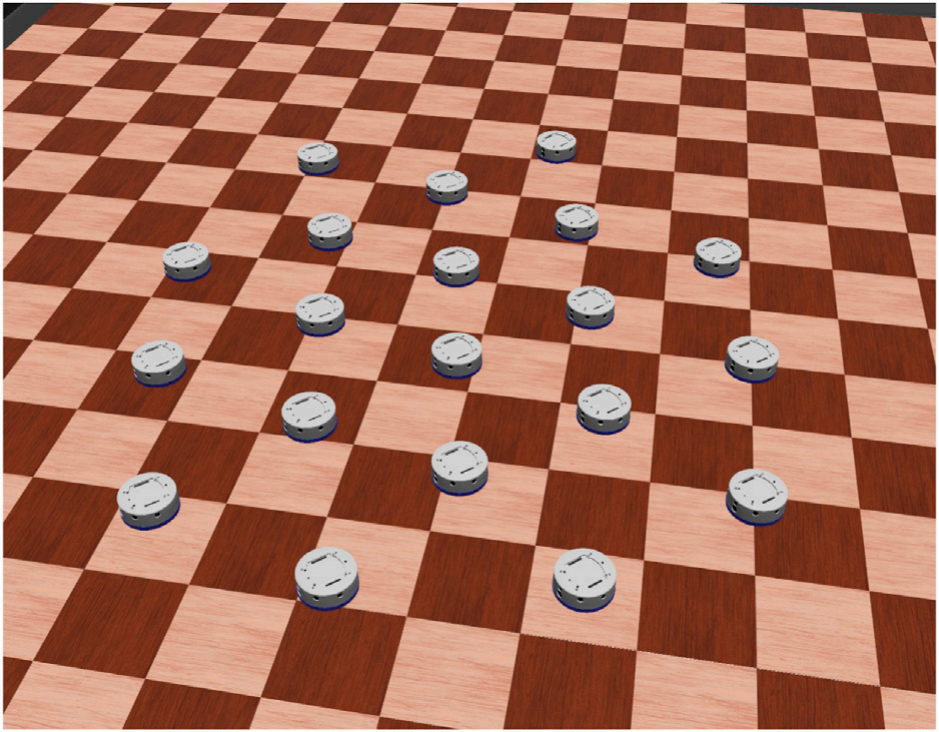
Submicroscopic simulation of a group of 20 Khepera IV robots.

Given our previous work in bridging the reality gap, as reported in Baumann and Martinoli (2021), we omitted real robot experiments here and considered the results of submicroscopic modeling to be the ground-truth. Thus, ground-truth experiments were conducted using simulated Khepera IV robots (see Soares et al. (2016)), equipped with a custom range and bearing board developed in Pugh et al. (2009). Khepera IV are differentially driven robots with a diameter of 14 cm and a maximal speed of ∼81 cm/s. The submicroscopic model of the MRS has been implemented in Webots Michel (2004), an open-source, high-fidelity robotics simulation platform. Sensors and actuators used in Webots were previously calibrated using dedicated experiments to match those of real Khepera IVs. For each submicroscopic simulation, the robots are randomly initialized within an area of size $\sqrt{N}$ x $\sqrt{N}$ m in an (considered to be) infinite arena. Figure 2 shows a flock of 20 robots in the Webots simulator.

### 2.1 Metrics

For assessing quantitatively the quality of the proposed microscopic models, we have decided to focus on the most interactive part of the collective movement, and thus decouple the trajectory followed by the flock as a whole from the maintenance of the flock over this trajectory as a result of individual members’ actions. To this purpose, we consider two different metrics, a fully continuous metric $M_{c}$ , defined in Eq. 3, and a discrete metric $M_{d}$ , defined in Eq. 4. Both metrics are based only on the closest three neighbors to obtain comparable metrics across different numbers of robots in the MRS. However, a sensitivity analysis on the impact of the number of neighbors used in the metric is provided in Section 6. $M_{c}$ corresponds to the average error with respect to the desired inter-agent distance D for the three closest neighbors of every robot. $M_{d}$ corresponds to the average number of neighbors which are within the “ideal range” ([D−ϵ,D+ϵ]) for the three closest neighbors of every robot.

$$M_{c}\left[k\right]=\frac{1}{N}\overset{N}{\underset{i=1}{\sum }}\frac{\sum \min_{3}\left(z_{i,j}\left[k\right]\right)-D}{3}$$ (3)

$$M_{d}\left[k\right]=\frac{1}{N}\overset{N}{\underset{i=1}{\sum }}\frac{\sum \phi \left(\min_{3}\left(z_{i,j}\left[k\right]\right)\right)}{3}$$ (4)

$$\text{with }\phi \left(z\right)=\left\{\begin{array}{cc} 1\; & \text{if }z\in \left[D-\epsilon ,D+\epsilon \right]\\ 0\; & \text{else} \end{array}\right.\text{, }j\in \left[1,N\text{\_}ngb\right]\text{, and }j\neq i$$

with *k* ∈ [0, *k*_*max*_] representing the index of the time step, with steps separated by a period *T* and *z*_*i*,*j*_ [*k*] the Euclidean distance between robots *i* and *j* at time step *k*. The numerical values used in this work are 50 ms for *T*, $k_{\max}=k_{120}=\frac{120\left[s\right]}{T}=2400$ and 0.15 m for *ϵ*.

To avoid biasing the metrics through initialization, we do not consider the first 20 s of a given run. That is:

$$\bar{M_{x}}=\frac{1}{k_{\max}-k_{20}}\overset{k_{\max}}{\underset{k=k_{20}}{\sum }}M_{x}\left[k\right]$$ (5)

with x denoting the type of the metric (c or d). These correspond to the metrics measured by both *μ*M-C and *μ*M-R and are thus the metrics used in our results. All plots reporting these metrics include vertical bars that indicate standard deviations. The number of repetitions of the experiments performed depends on the model used: experiments using the *μ*M-A and submicroscopic model are repeated 20 times; *μ*M-C experiments are repeated 40 times and *μ*M-R experiments are repeated 80 times.

### 2.2 Microscopic models

In the following sections, we will introduce the three microscopic models used in this work, starting with the more accurate ***μ*** **M-A**, a spatial microscopic model keeping track of the absolute position of the agents and leveraging widely used multi-agent simulation principles. We then present ***μ*** **M-C**, a canonical probabilistic microscopic model which considers a discretized spatial representation of the neighborhood of every agent. Finally, we introduce our novel ***μ*** **M-R**, which maintains a continuous spatial representation of the relative distances of the neighboring teammates from a representative agent in the flock.

Each model is detailed, and its performance is reported, in its respective section. For both *μ*M-R and *μ*M-C, we further discuss geometric reasoning that enables us to reduce the number of experiments needed to calibrate their respective parameters.

## 3 The absolute microscopic model *μ*M-A

**FIGURE 3:**
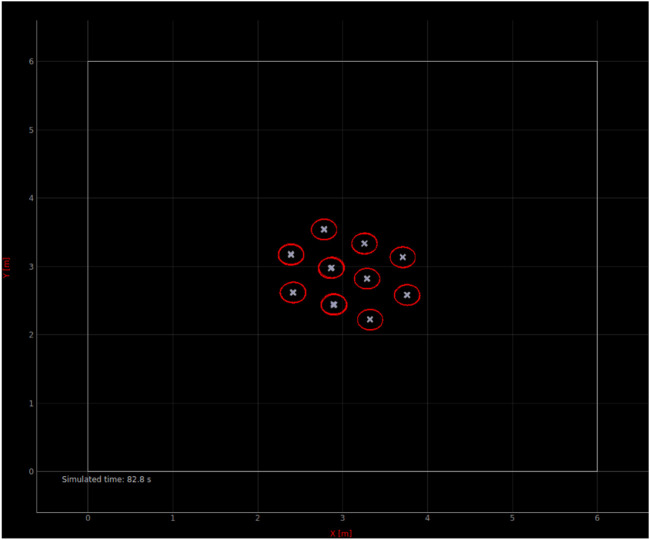
Screenshot of our custom spatial microscopic simulator implementing our *μ*M-A.

A fully spatial microscopic model keeping track of the absolute position of each agent in the group is a highly intuitive way to model collective movements; an example of implementation of the corresponding microscopic simulator is given in Figure 3.

### 3.1 Modeling choices and structure

This custom-implemented simulator resembles a submicroscopic simulator with a few crucial simplifications at the individual robot level:

• Every type of discrete sensor is aggregated into an omnidirectional sensor, one for each sensing modality.
• Robots are represented by point masses, whose pose is indicated by the “x” in Figure 3 (the heading is not visualized). The red outlines of the robots are visual aids only.
• Robot kinematics can be accurately represented. In our case, we use forward kinematic equations for differential driven robots. The impact of modeling kinematics is discussed in Section 3.2.
• In contrast to submicroscopic simulators, friction, wheel slip, body-to-body collisions, etc. are neglected.

**FIGURE 4:**
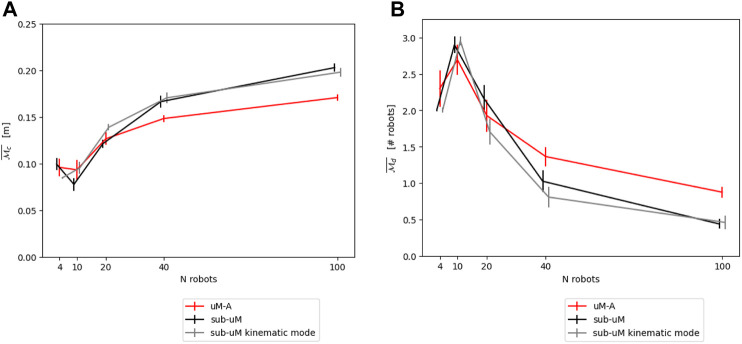
**(A)** Comparison between *μ*M-A (using kinematics) and the submicroscopic model using the continuous metric $\bar{M_{c}}$ . **(B)**. Comparison between *μ*M-A (using kinematics) and the submicroscopic model using the discrete metric $\bar{M_{d}}$ .

Figures 4A,B show the resulting modeling output compared to the submicroscopic ground-truth, as well as a submicroscopic model using kinematics instead of full physics. This latter is interesting as most commonly used submicroscopic simulators provide such a kinematics mode, which is thus trivial to apply. Note that in all figures, the dot positions of the data points of the different models are slightly offset along the horizontal axis to increase the readability of the figures.

### 3.2 Parameter calibration

**FIGURE 5:**
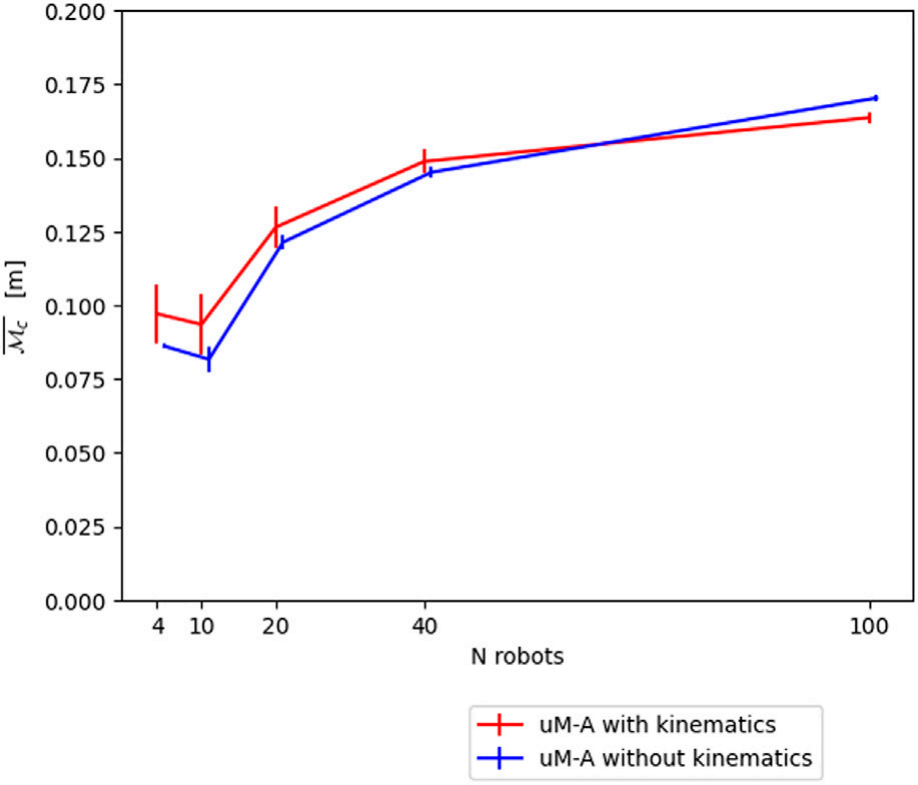
Impact of using accurate kinematics in *μ*M-A for our flocking model using the continuous metric $\bar{M_{c}}$ .

In order to calibrate *μ*M-A, all represented parameters and coefficients are matched as well as possible to submicroscopic experiments. For example, the amount of noise of the omnidirectional range and bearing sensor is set to the average noise level of the corresponding submicroscopic sensors. The only less trivial aspect is the calibration of the motor-command-to-robot-displacement chain. Since motors, gears, etc. are omitted from the robot model in *μ*M-A, we approximated this complete (and complex) chain with a single linear coefficient, which is again calibrated based on submicroscopic experiments. It is worth highlighting that the submicroscopic model has previously been calibrated in an equivalent way to the physical system, though naturally with a much richer set of parameters. Another important aspect of the calibration of *μ*M-A is the decision to accurately model the vehicles’ kinematics, as its impact depends on the scenario and metric considered. Figure 5 shows the difference for the *μ*M-A model, using $M_{c}$ . A Mann-Whitney-U-Test has been performed, using 20 runs each, to determine whether this difference is statistically significant. The *p*-values obtained for the continuous metric are [ < 0.001, 0.026, 0.116, <0.001, <0.001] for [4, 10, 20, 40, 100] robots, respectively. Therefore, we can conclude that the impact of accurately simulating kinematics for this case study is statistically significant, and we will thus only use *μ*M-A with kinematics for the remainder of this work, even though the discrepancy between the submicroscopic model and *μ*M-A is larger than this difference. It should be noted that the observed errors might be acceptable for a specific modeling purpose, in which case the kinematics could be omitted, resulting in a ∼25% reduction of the computational cost in our implementation.

## 4 The canonical microscopic model *μ*M-C

Canonical probabilistic microscopic models, *μ*M-C, as introduced in Martinoli et al. (2004), are built in a bottom-up fashion, leveraging the states of the control algorithm to define the state space of the model. The transitions between these states subsequently correspond to interactions between a robot and its environment or other robots. However, in contrast to both *μ*M-A and *μ*M-R, interactions are represented probabilistically only, defined through time delays and encountering probabilities. In order to define a *μ*M-C, the following main steps are necessary:

• Separation of the group trajectory from the interactions within the group;
• Decision on distinct, discrete states to represent the state-space of the model;
• Definition of metrics computable with the tracked states;
• Calibration of the transition rates between the states using knowledge of the underlying controller, geometrical reasoning, or by measuring them in a more accurate model.

### 4.1 Modeling choices and structure

**FIGURE 6:**
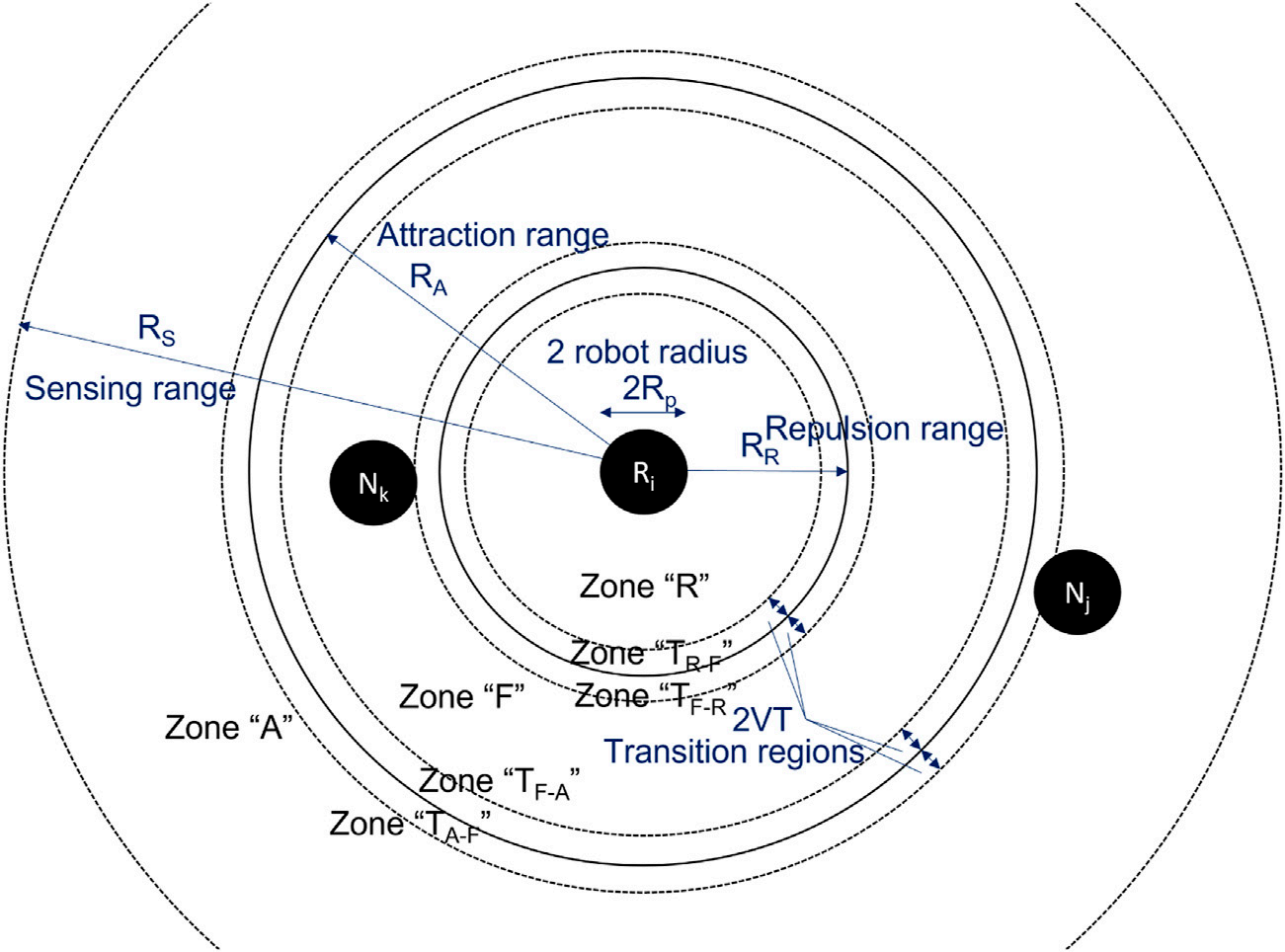
Spatial discretization used in *μ*M-C.

In our case study, the selected control algorithm (see Section 2) does not have distinct states. We therefore revert to a spatial discretization similar to the work of Winfield et al. (2008). Figure 6 shows an illustration of this discretization. Using the point of view of robot *R*_*i*_, three different circular zones are defined around it: zone “R”–repulsion, zone “F”–flocking, and zone “A”–attraction. This is based on the intuition that the prominent pairwise behavior for a very close neighbor is repulsion, and the prominent behavior for a far away neighbor is attraction. In the middle, that is for a neighbor distance between D−ϵ and D+ϵ , no predominant behavior can be found. Therefore, this zone is considered the “optimal” flocking zone, as illustrated in Figure 1. Using predominant behaviors, we can thus obtain distinct control states which we subsequently leverage to build our model as shown in the following. It should be noted that the definition of zone F must match the definition of the ideal flocking distance in metric $M_{d}\left[k\right]$ for *μ*M-C to be meaningful. More generally, the metric of interest needs to be computable as a function of the model states.

Based on the three zones R, F and A, we can compute the exact number of neighbors in each zone for each robot *R*_*i*_ in the flock using the following difference equations, capturing the robot balance across zones:

$$\begin{array}{cc} N_{R}\left[k+1\right] & =N_{R}\left[k\right]-\Delta_{R-F}\left[k\right]+\Delta_{F-R}\left[k\right]\\ N_{F}\left[k+1\right] & =N_{F}\left[k\right]+\Delta_{R-F}\left[k\right]-\Delta_{F-R}\left[k\right]+\Delta_{A-F}\left[k\right]-\Delta_{F-A}\left[k\right]\\ N_{A}\left[k+1\right] & =N_{A}\left[k\right]-\Delta_{A-F}\left[k\right]+\Delta_{F-A}\left[k\right] \end{array}$$ (6)

where $N_{R}\left[k\right]$ , $N_{F}\left[k\right]$ , $N_{A}\left[k\right]$ are the number of neighboring robots at time step *k* within the repulsion, flocking, and attraction zones, respectively.

Each Δ is the sum of neighbors that transition at time step *k*:

$$\begin{array}{cc} \Delta_{R-F}\left[k\right] & =\underset{N_{R}\left[k\right]}{\sum }P\left(p_{R-F}\right)\\ \Delta_{F-R}\left[k\right] & =\underset{N_{F}\left[k\right]}{\sum }P\left(p_{F-R}\right)\\ \Delta_{F-A}\left[k\right] & =\underset{N_{F}\left[k\right]}{\sum }P\left(p_{F-A}\right)\\ \Delta_{A-F}\left[k\right] & =\underset{N_{A}\left[k\right]}{\sum }P\left(p_{A-F}\right) \end{array}$$ (7)

with $P\left(p_{i}\right)=\left\{\begin{array}{cc} 0\; & |rng\geq p_{i}\\ 1\; & |rng<p_{i} \end{array}\right.$ , where *rng* is a randomly generated number $\in \left[0\right.,1\left[\right.$ .

### 4.2 Parameter calibration

**FIGURE 7:**
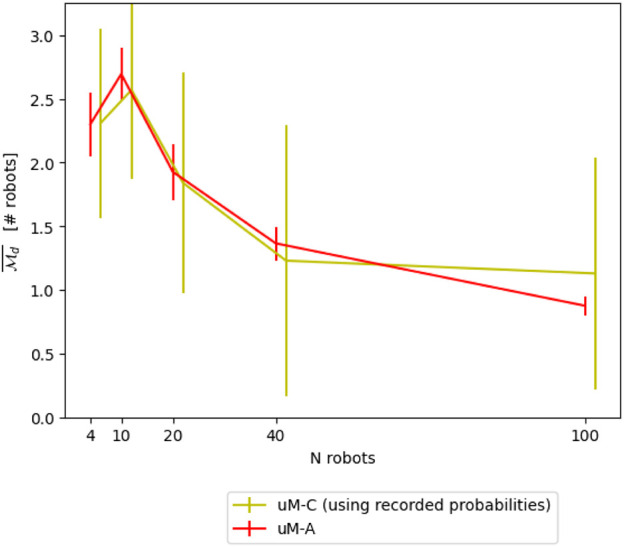
Comparison between *μ*M-C and *μ*M-A using recorded probabilities of *μ*M-A for the $\bar{M_{d}}$ metric.

A very intuitive way to numerically define the probabilities *p* is to measure them based on recorded experiments carried out with a more accurate and typically richer model. Table 1 reports the probabilities obtained for different numbers of robots using the *μ*M-A for an 8-shape trajectory. Figure 7 shows the resulting modeling performance compared μM-A, using μM-A as ground-truth for calibration.

**TABLE 1:** Recorded *μ*M-C transition probabilities obtained through *μ*M-A simulations.

	*N* = 4	*N* = 8	*N* = 10	*N* = 20	*N* = 40	*N* = 100
*p*_*R*−*F*_	0.1043	0.0941	0.0887	0.0444	0.0220	0.0118
*p*_*F*−*R*_	0.0001	0.0024	0.0051	0.0120	0.0096	0.0063
*p*_*F*−*A*_	0.0018	0.0019	0.0019	0.0045	0.0066	0.0056
*p*_*A*−*F*_	0.0053	0.0017	0.0013	0.0012	0.0008	0.0003

#### 4.2.1 Geometrically defined transition probabilities

While basing the transition probabilities directly on dedicated, more accurate experiments results in modest modeling errors, this procedure significantly limits the analytical impact of the abstraction effort originally motivating many of the modeling choices of *μ*M-C. Additionally, given the spatiality, respectively, non well-mixedness of the system, the transition probabilities change non-linearly with the number of robots *N*, as illustrated by Table 1. This in turn means that dedicated experiments are necessary for every *N* of interest, further limiting the impact of the model. In this section, we therefore explore geometrical reasoning in combination with system identification techniques as a valid alternative to properly estimate the transition probabilities, for all possible number of robots *N*.

As in Winfield et al. (2008), we can define “transition zones”, that is, regions where the transition from one zone to another *can* occurs in a time step of duration *T* for a given (average) robot speed *V*. These regions are indicated as zones *T*_*X*−*Y*_ in Figure 6. The conditional probability of being in one of these zones given one of the three main zones (R, F, A) can be calculated geometrically:

$$\begin{array}{cc} P_{T_{F-R}}|F & =\frac{{\left(R_{R}+2VT\right)}^{2}-R_{R}^{2}}{R_{A}^{2}-R_{R}^{2}}\\ P_{T_{F-A}}|F & =\frac{{R_{A}}^{2}-{\left(R_{A}-2VT\right)}^{2}}{R_{A}^{2}-R_{R}^{2}}\\ P_{T_{A-F}}|A & =\frac{{\left(R_{A}+2VT\right)}^{2}-{\left(R_{A}\right)}^{2}}{R_{S}^{2}-R_{A}^{2}}\\ P_{T_{R-F}}|R & =\frac{{R_{R}}^{2}-{\left(R_{R}-2VT\right)}^{2}}{R_{R}^{2}-R_{p}^{2}} \end{array}$$ (8)

with *R*_*R*_, *R*_*A*_ and *R*_*S*_ the radii of the respective zones, as illustrated in Figure 6. Each probability corresponds to the ratio of the corresponding transition zone’s size over the size of the current zone. Intuitively, this corresponds to the probability of an agent ending up in the transition zone, given the current zone. This assumes that the agents are “hopping”, that is, not following a trajectory but stochastically changing positions. This introduction of hopping is a key simplification necessary to achieve the non-spatialness of the microscopic model as introduced in Martinoli et al. (2004), however, it requires the system to be well-mixed in the region of interest (over the whole arena in case of an enclosed one), resulting in a roughly uniform distribution of the agents. In contrast to the previous contribution, here, the robots are hopping only within their zone instead of globally. Accordingly, only well-mixedness within a given zone is assumed. This more granular representation of spatiality enables the model to represent situations with a lower amount of stochasticity. Using these probabilities, we can rewrite Eq. 7 as

$$\begin{array}{cc} \Delta_{R-F}\left[k\right] & =N_{R}\left[k\right]\left[P_{T_{R-F}}|R\right]\Gamma_{R-F}\\ \Delta_{F-R}\left[k\right] & =N_{F}\left[k\right]\left[P_{T_{F-R}}|F\right]\Gamma_{F-R}\\ \Delta_{F-A}\left[k\right] & =N_{F}\left[k\right]\left[P_{T_{F-A}}|F\right]\Gamma_{F-A}\\ \Delta_{A-F}\left[k\right] & =N_{A}\left[k\right]\left[P_{T_{A-F}}|A\right]\Gamma_{A-F} \end{array}$$ (9)

where each Γ represents the probability that a neighbor will actually change zone from the respective transition regions.

#### 4.2.2 Fine-tuning transition probabilities with system identification

Assuming neighbors are moving randomly, the transition probability of Eq. 9 would be roughly 50%. However, in our case, the robot control is highly deterministic. Therefore, a more fine-tuned calibration is required. Similarly to Correll and Martinoli (2006), we leverage to this purpose a system identification method (Ljung (1998)) consisting of the following three basic steps:

1) Gathering of ground-truth data using dedicated perturbation experiments;
2) Creation of a candidate model;
3) Minimization of the difference between the candidate model and the ground-truth data using optimization.

In order to gather enough ground-truth data for the size of the parameter vector to be calibrated (and thus avoiding an underdetermined system), four sets of perturbation experiments of *L* = 20 runs each for *N* = 4, 10, 20, 100 number of robots were carried out using the next-most accurate model, *μ*M-A.

The candidate model has been described before, using Eqs. 8 and 9. The initial conditions are defined using a purely geometric approach:

$$N_{R}\left[0\right]=\frac{R_{R}^{2}}{R_{S}^{2}}$$ (10)

$$N_{F}\left[0\right]=\frac{{\left(R_{A}-R_{R}\right)}^{2}}{R_{S}^{2}}$$ (11)

$$N_{A}\left[0\right]=\frac{{\left(R_{S}-R_{A}\right)}^{2}}{R_{S}^{2}}$$ (12)

It is worth noting that, given the linearity of the model, we could also have expressed it in matrix notation which does not, however, increase its readability and is thus omitted here.

**FIGURE 8:**
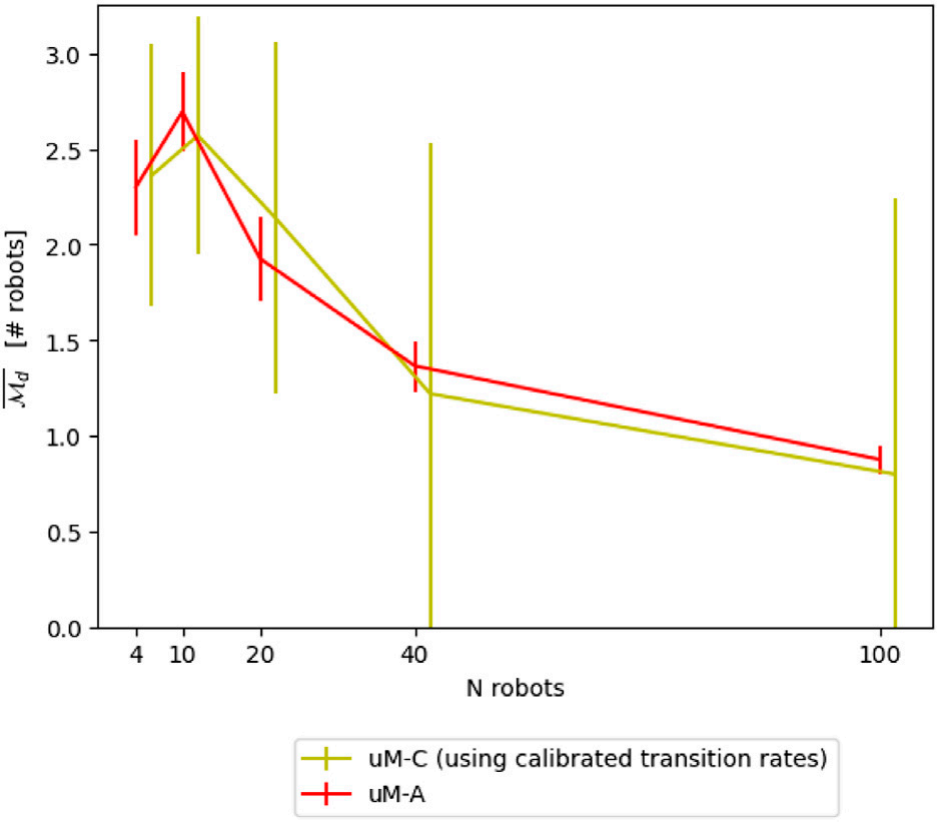
Comparison between *μ*M-C and *μ*M-A using geometrical reasoning and transition probabilities obtained through system identification.

We want to minimize the prediction error of the model estimate $\hat{\bar{M_{d}}}\left(\theta ,N\right)$ compared to ground-truth perturbation experiments $\bar{M_{d}}\left(N\right)$ by choosing the optimal parameter set *θ*_*o*_ = {Γ_*R*−*F*_, Γ_*F*−*R*_, Γ_*F*−*A*_, Γ_*A*−*F*_}. That is, we want to solve the following optimization problem:

$$\theta_{o}=\underset{\theta }{\mathrm{arg min}}\left(\underset{N_{i}\in N}{\sum }\overset{L}{\underset{l=1}{\sum }}{\left(\hat{\bar{M_{d}}}\left(\theta \right)-\bar{M_{d}}\right)}^{2}\right)$$ (13)

with *N*_*i*_ the number of robots in an experiment. Using the Nelder-Mead optimization of SciPy with *N*_*i*_ ∈ [4, 10, 20, 40] results in the transition probabilities as indicated in Table 2. The resulting modeling performance is shown in Figure 8. The optimization has been performed using a maximum of 2000 function calls and with a tolerance of 0.0001 for early convergence.

**TABLE 2:** Transition probabilities for *μ*M-C obtained through geometrical reasoning and system identification.

Γ_*R*−*F*_	Γ_*F*−*R*_	Γ_*F*−*A*_	Γ_*A*−*F*_
0.91	0.09	0.03	0.74

## 5 The relative microscopic model *μ*M-R

The relative microscopic model, ***μ*** **M-R**, maintains a continuous spatial representation of the relative distances of the neighboring teammates from a representative agent in the flock. As such, the level of abstraction of this model is situated in between *μ*M-A and *μ*M-C. In order to define a *μ*M-R, the following steps are required:

• Separation of the group trajectory from the interactions within the group;
• Determination of the distribution of an average neighborhood of a robot within the group using geometrical reasoning, system identification or calibration experiments using a more accurate model;
• Re-formulation of the metric(s) to require only the point of view of a single (representative) robot.

### 5.1 Modeling choices and structure

*μ*M-R is a spatial model; as such the robots are represented in a two dimensional space. However, the model is fully aligned with the definition of the metrics. That is, the deterministic trajectory of the group is omitted, focusing instead on the local interactions. The stochastic interactions between flock members are modeled using the same principles as for *μ*M-A (aggregated sensors, with or without kinematics, neglected physics). However, to further reduce model complexity, we do not model the complete MRS, but only a representative robot surrounded by an averaged neighborhood representative of the whole flock. For this, we initialize *N*_*ngb* neighbors around a robot *R*_*i*_ in a randomized fashion, as described in the next subsection. Subsequently, those neighbors are considered static in a global reference, with only the robot *R*_*i*_ moving according to the action function defined in Eq. 2. To minimize the bias of the metrics through the initialization of the neighbors, we consider only the metric of the central (non-static) robot. Therefore, the metrics of Eqs. 3 and 4 are changed to:

$$\begin{array}{cc} M_{c}\left[k\right] & =\frac{\sum \left(\min_{3}\left(z_{i,j}\left[k\right]\right)-D\right)}{3}\\ M_{d}\left[k\right] & =\frac{\sum \phi \left(\min_{3}\left(z_{i,j}\left[k\right]\right)\right)}{3}\\ \text{with } & j\in \left[1,N\text{\_}ngb\right] \end{array}$$ (14)

It is worth noting, that the speedup between this model and *μ*M-A is a function of the ratio between the number of robots *N* and the local neighborhood *N*_*ngb*, namely if the complexity of *μ*M-A is $O\left(N^{2}\right)$ , the complexity of *μ*M-R is O(N_ngb) .

### 5.2 Parameter calibration

One of the critical calibration aspects of this model is related to the construction of an averaged neighborhood representative of the whole flock. Similar to the parameter calibration of the *μ*M-C, we present in the following two techniques for defining the averaged neighborhood, one exploiting data gathered with a more faithful model and one leveraging geometrical reasoning.

#### 5.2.1 Averaged neighborhood defined with experimental data

**FIGURE 9:**
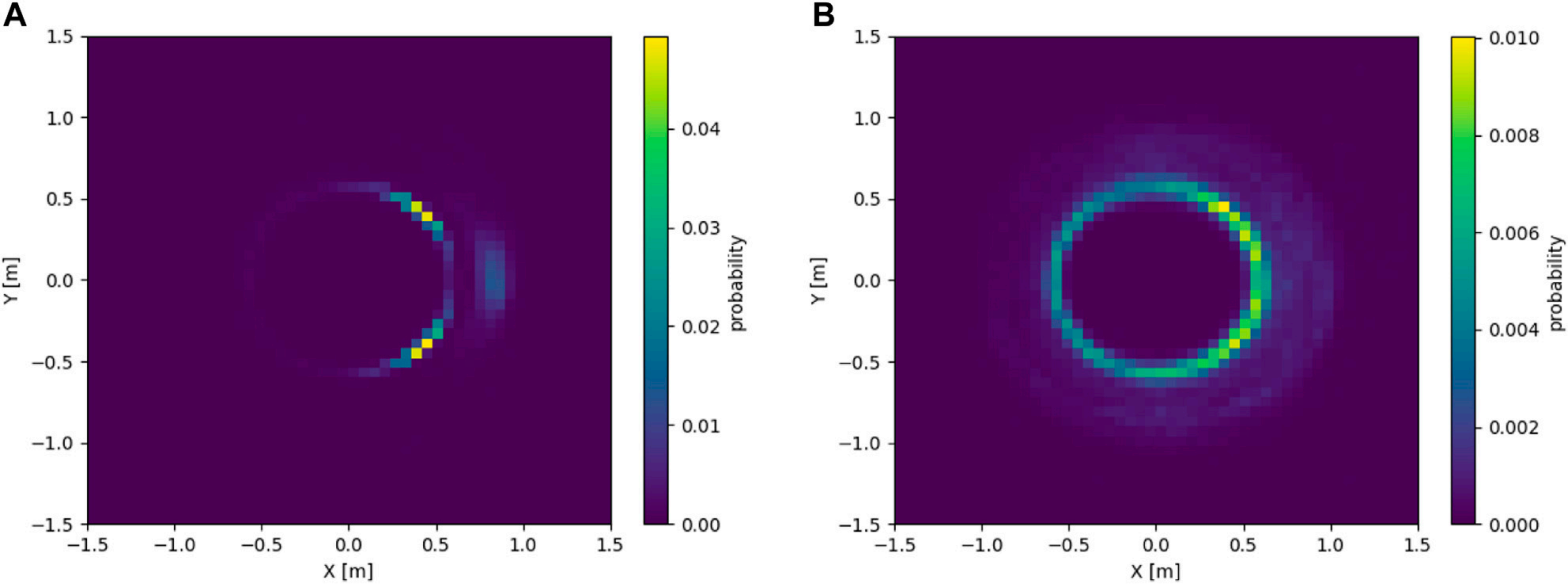
**(A)** PDF of neighbor positions for a static flock of four robots (simulated using *μ*M-A), from the point of view of a robot placed at (0,0) facing east. **(B)** PDF of neighbor positions for a flock of four robots tasked to follow an 8-shape trajectory (simulated using *μ*M-A), from the point of view of a robot placed at (0,0) facing east.

Based on recorded positions using the next-most accurate model, *μ*M-A (using 20 experiments in our case), similar to the work of Zhang et al. (2008), it is possible to establish Probability Density Functions (PDFs) of the position of neighbors for a given flock size and flock trajectory. This spatiotemporal aggregation includes the positions of all neighboring robots of each robot *R*_*i*_ in the flock for every time step *k* ∈ [0, *k*_max_]. Figure 9A shows an example of a PDF for a static flock of four robots. In Figure 9B, the flock is tasked to follow an 8-shape trajectory. The similarities between the two PDFs are apparent, but so are the differences, demonstrating that the neighboring positions depend on the trajectory assigned to the flock. Although not demonstrated in this work, we expect the presence of obstacles to have a similar effect on the PDF.

Unfortunately, PDFs are not directly usable for initializing neighboring robots in *μ*M-R, as once a neighbor is placed, its position influences the PDF of the remaining neighbors, as in reality, the neighboring robots are also following the same flocking control law and are thus also coordinated amongst each other. Indeed, drawing positions without replacement from the PDF does not take this aspect into account. It is therefore necessary to cluster positions, where each cluster corresponds to the position of *one* neighbor. This can be achieved, for example, through Gaussian Mixture Models (GMMs).

**FIGURE 10:**
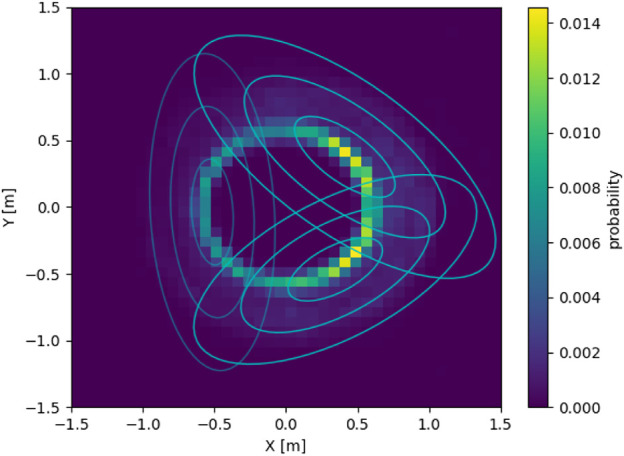
A GMM overlayed on the PDF of neighbor positions for a flock of four robots following an 8-shape (simulated using *μ*M-A), from the point of view of a robot placed at (0,0) facing east.

Using the same aggregated experimental data as before, we can fit a GMM on the data rather than just generating a PDF. In this work, the GMMs have been created using the *Python* library sklearn. The number of clusters (*K*) has been varied from *N*_*ngb*, the number of neighbors according to Eq. 18, to *N*, the number of robots in the flock. For each *K*, four initializations of K-means of the cluster have been done with a maximum of 100 iterations each. For each flock size, only the GMM with the highest silhouette score (Rousseeuw (1987)) has been retained. Figure 10 shows the resulting GMM for the situation previously considered of a flock of four robots tasked with following an 8-shape trajectory.

In order to initialize a neighbor in *μ*M-R, one of the Gaussian distributions of the GMM is drawn randomly (without replacement), taking into account the respective weights of the Gaussian components in the GMM. The neighboring robot’s position is then drawn from the selected Gaussian distribution.

The number of neighbors *N*_*ngb* is calculated using geometrical reasoning within an ideal flock. That is, a flock where the distance between every first-order neighbor is precisely the desired inter-robot distance D , leading to a hexagonal lattice shape of the flock. Taking into account the sensing range *R*_*w*_ = 1.5 m of the range and bearing sensor endowing each robot, we can define the total number of neighbors as follows, following closely the calculation and notation of appendix A of Winfield et al. (2008):

$$N\text{\_}ngb^{\max}=N_{0}+2\overset{l_{\max}}{\underset{l=1}{\sum }}N_{l}-1$$ (15)

using

$$N_{l}=\frac{\sqrt{4R_{w}^{2}-3D^{2}l^{2}}}{D}+1\text{, with }l\in \left[0,l_{\max}\right]$$ (16)

and

$$l_{\max}<\frac{2R_{w}}{\sqrt{3}D}$$ (17)

where $l_{\max}$ is the number of “rows” of neighboring robots within the the sensing range, and $N_{l}$ the number of robots in row l . The number of neighbors *N*_*ngb* can finally be defined using the total number of robots *N* as

$$N\text{\_}ngb=\min\left(N\text{\_}ngb^{\max},N-1\right)$$ (18)

**FIGURE 11:**
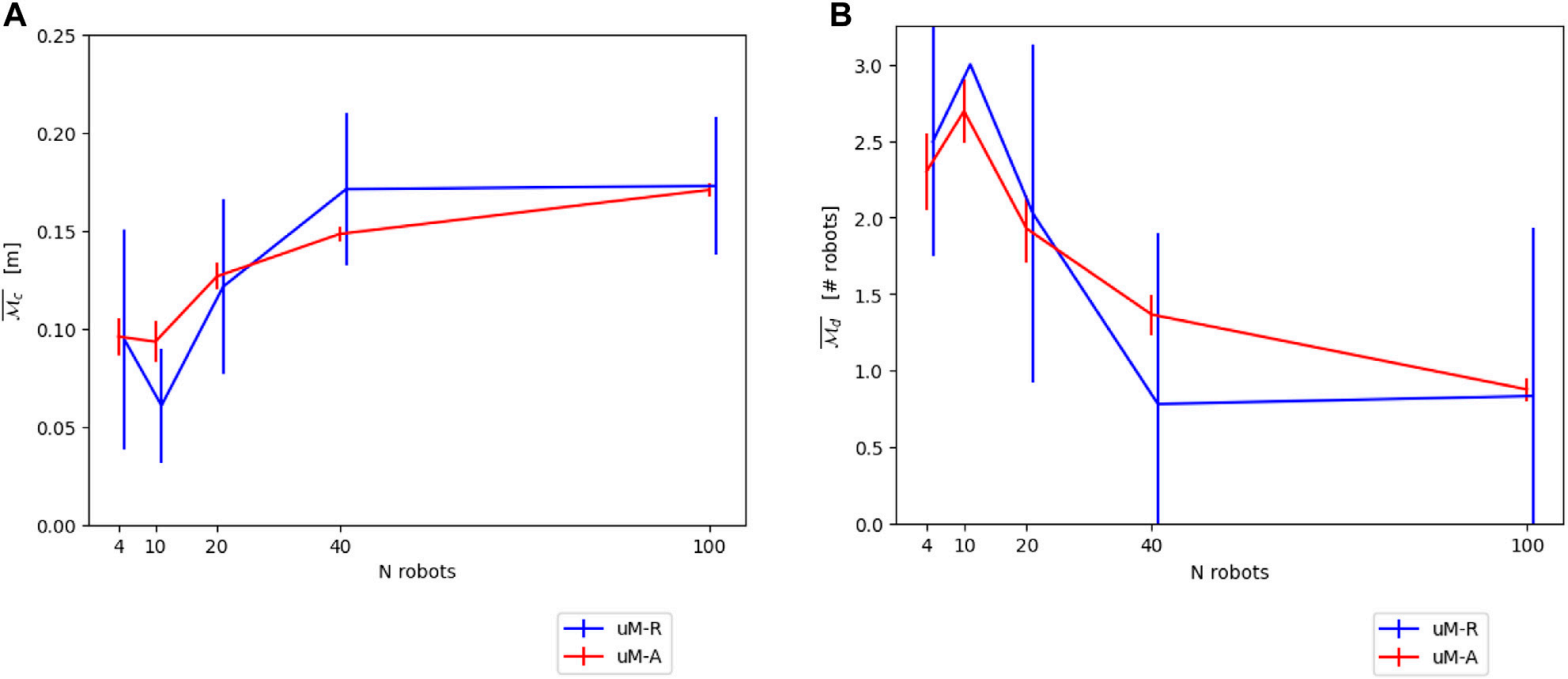
**(A)** Comparison between *μ*M-A and *μ*M-R using a GMM-defined averaged neighborhood on the continuous metric $\bar{M_{c}}$ . **(B)** Comparison between *μ*M-A and *μ*M-R using a GMM-defined averaged neighborhood on the discrete metric $\bar{M_{d}}$ .

The final performance of *μ*M-R compared to *μ*M-A is shown in Figures 11A,B.

#### 5.2.2 Averaged neighborhood defined with geometrical reasoning

Similar to the *μ*M-C, while constructing the averaged neighborhood on data gathered with more faithful models results in good performance, this implies the need for dedicated prior experiments to conduct this calibration. An intuitive manner to avoid this is to base the neighbor positions on geometrical reasoning. We based our approach on an ideal flock assuming a hexagonal lattice shape. Using the underlying robot control law in Eq. 2, we can calculate the distance *d* to the first neighbors of a robot *R*_*i*_ as the distance where the sum of all pairwise controls is 0:

$$0=\overset{N\text{\_}ngb}{\underset{j=1}{\sum }}u\left(z_{i,j}\right)=\overset{N\text{\_}ngb}{\underset{j=1}{\sum }}\left(1-\frac{D^{7}}{z_{i,j}^{7}}\right)$$ (19)

**FIGURE 12:**
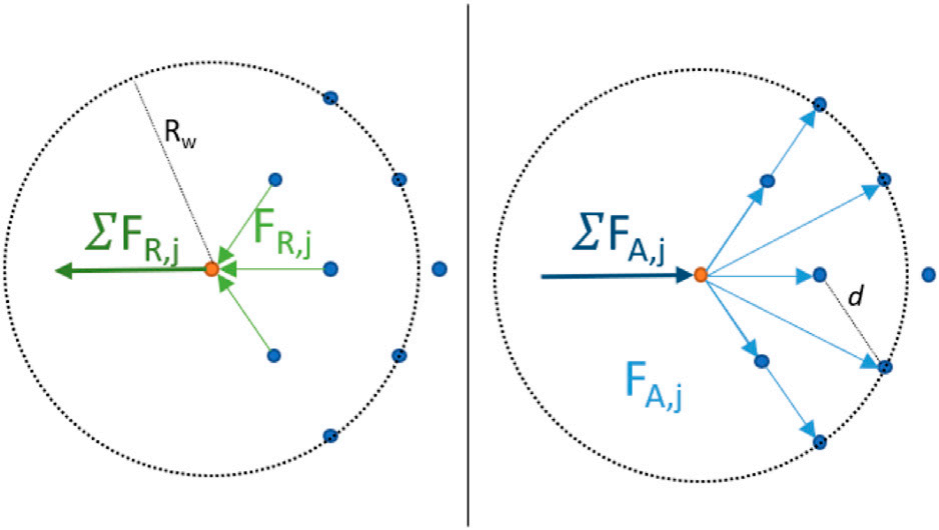
A simplified illustration of the forces acting on a robot *R*_*i*_ (orange). *F*_*A*,*j*_ corresponds to the attracting force due to neighbor *j* and *F*_*R*,*j*_ to the corresponding repulsive force of neighbor *j*.

We can split the forces in attractive (*F*_*A*_) and repulsive forces (*F*_*R*_) and neglect for the latter all but first-order neighbors (which are situated at *z* = *d*), as illustrated in Figure 12. Considering (without loss of generality) the situation as illustrated in Figure 12, we obtain:

$$0=\overset{N\text{\_}ngb}{\underset{j=1}{\sum }}F_{A,j}-\overset{N\text{\_}ngb}{\underset{j=1}{\sum }}F_{R,j}\approx \frac{N\text{\_}ngb}{\pi }-\left(1+\cos\left(\pi /3\right)+\cos\left(-\pi /3\right)\right)\frac{D^{7}}{d^{7}}$$ (20)

which gives us

$$d=\sqrt[{7}]{\frac{D^{7}\left(1+\cos\left(\pi /3\right)+\cos\left(-\pi /3\right)\right)}{N\text{\_}ngb/\pi }}$$ (21)

Note that we consider a robot at the very edge of the flock for this calculation, as, for a robot in the center, assuming a “perfect” flock with equal distances between all first-order neighbors, attraction and repulsion from all directions will cancel each other out. Therefore, the distance between robots *d* is given solely through the control of the outermost robots.

Considering again Eq. 18 for the number of neighbors given a flock size, we realize that it depends in turn on the inter-robot distance D . By replacing D with *d* obtained using Eq. 21, we obtain a set of inter-dependent equations which can be solved for example using numerical methods. In this work, we used the classical Nelder-Mead method (Nelder and Mead (1965)) with *d*_0_ = 0.5 m to obtain d as a function of the flocksize *N*.

**FIGURE 13:**
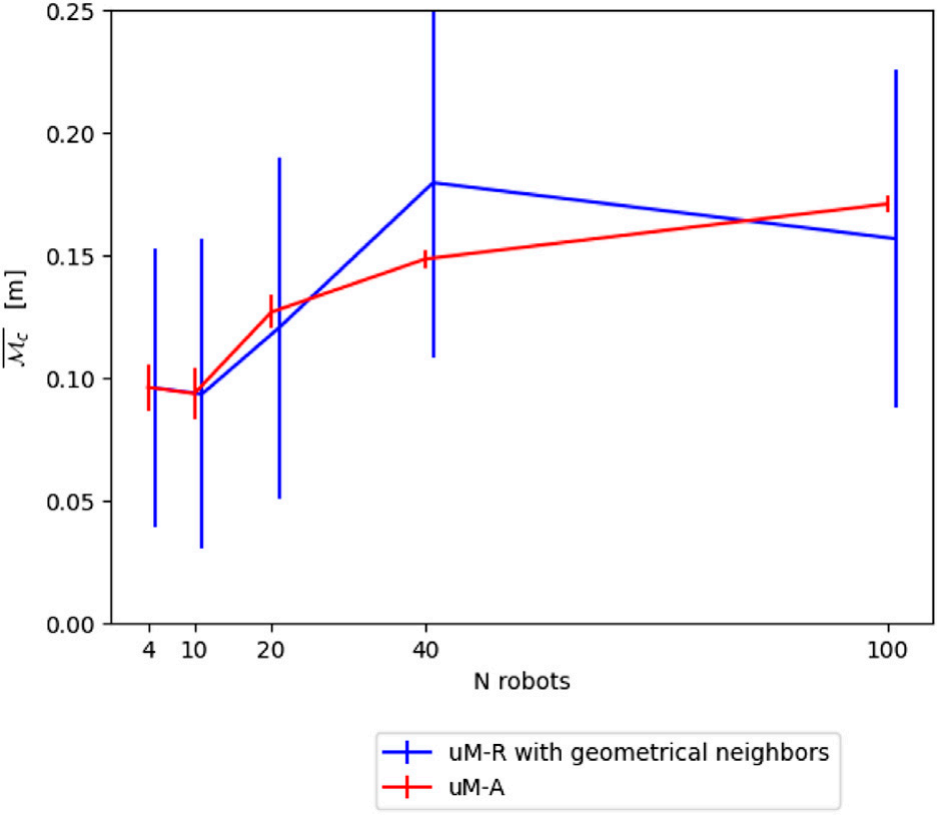
Comparison between *μ*M-A and *μ*M-R using geometrical reasoning to construct the averaged neighborhood on the continuous metric $\bar{M_{c}}$ .

Given the expected inter-robot distance *d*, as well as the clean lattice format of our flocking, we can easily initialize neighbors in the *μ*M-R model in a hexagonal lattice with an edge length of *d*, without recurring to any construction of a GMM-based probabilistic landscape as in Section 5.2.1. In order to provide a more realistic simulation, additional Gaussian noise can be added to the neighboring positions. Figure 13 shows the resulting performance for *μ*M-R using a Gaussian noise with *σ* = 0.2 in comparison to *μ*M-A.

## 6 Overall comparison and discussion

**FIGURE 14:**
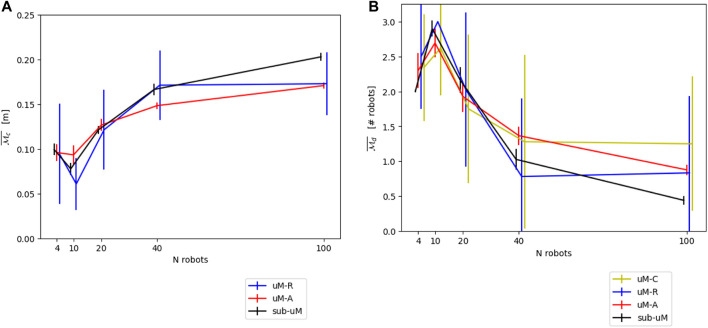
**(A)** Overall comparison between the different models for the continuous metric $\bar{M_{c}}$ . **(B)** Overall comparison between the different models for the discrete metric $\bar{M_{d}}$ .

Figures 14A,B show the resulting modeling performance for both metrics $\bar{M_{c}}$ and $\bar{M_{d}}$ . Both *μ*M-R and *μ*M-C reported here correspond to the modeling version using parameters calibrated with prior experiments using *μ*M-A. However, as shown in Sections 4.2.1 and 5.2.2, using geometrical reasoning results in similar performances. Since *μ*M-C can only produce results for the discrete metrics $\bar{M_{d}}$ , this microscopic model variant has been omitted in the comparison on the continuous metric $\bar{M_{c}}$ .

The fact that the *μ*M-A matches well with the submicroscopic simulator for both metrics confirms that the common practice of using (custom) spatial microscopic simulators is indeed adequate for metrics of interest similar to the ones used here. It is worth stating, however, that *μ*M-A does not represent hardware and thus hardware-related parameters. While it is possible to include those in the simulation, the *μ*M-A will, in this case, degenerate sooner or later to a submicroscopic model. Given the availability of high-quality open-source submicroscopic simulators, we believe that such an exercise is not needed for most applications.

As expected, both calibrated models, *μ*M-R and *μ*M-C, closely match the calibration baseline. The accuracy obtained is, in our view, an impressive demonstration of the capabilities of the modeling structures of both *μ*M-C and *μ*M-R. However, we note that *μ*M-R includes a significant standard deviation due to the randomization inherent to the use of an averaged neighborhood constructed probabilistically. Moreover, we showed that, while the parameters of this models (e.g, transition probabilities, GMMs) can be calibrated with dedicated data gathered using more faithful models, geometrical reasoning can help in further reducing the calibration effort and promoting thus additional analytical insight in the dynamics of the MRS.

One of the key reasons for leveraging modeling instead of high-fidelity simulations or even real robot experiments is the speedup in comparison to wall-clock time. As a result, we report in Table 3 the speedups obtained using our implementations. The speedup is defined as the ratio between the time that would be needed to run the experiment with real robots vs. the time needed to run the experiment using a given model, on a given hardware. In our case, a real robot experiment takes 120s independently of the number of robots involved. For four robots, sub-*μ*M takes ∼4.8 s, resulting thus in a speedup of 25. All experiments have been conducted on an Intel^®^ Core™ i9-11900 CPU @2.50 Hz processor with an NVIDIA GeForce RTX 3060. It is worth noting that the sub-*μ*M is implemented in C, while all other models have been implemented in *Python*. Nevertheless, *μ*M-A uses a limited amount of Cython to speed up the simulation. Additionally, the models in this work use one thread only. However, all of them could further be optimized for computational speed through parallelism. As a result, the speedup values reported in Table 3 should be taken as indicator of the order of magnitude for each model rather than precise speedup values, as the latter will always depend on the concrete implementation choices. Nonetheless, it is interesting to realize that, as soon as robot numbers increase, a submicroscopic simulation cannot provide a high amount of speedup (for one robot, speedups of up to 50x can be observed). When using kinematic mode instead of full physics, a slight computational gain can be observed. However, typically no additional simplifications are made and, depending on the implementation of the simulator, less work is offloaded to GPUs, which limits the impact of the kinematic mode on the computational cost. Using a custom *μ*M-A results in an increased speedup by one order of magnitude, with *μ*M-R increasing it further with an even more significant impact for larger numbers of robots. The most extreme speedups of four orders of magnitude are achieved by the *μ*M-C.

**TABLE 3:** Simulation speedups recorded in our implementations in comparison to wall-clock time for different flock sizes *N*.

*N*	Sub-*μ*M	Sub-*μ*M (kin)	*μ*M-A	*μ*M-R	*μ*M-C
4	x 25	x 28	x 200	x 239	x 15,000
10	x 8	x 9	x 67	x 146	x 8,500
20	x 2	x 2.3	x 26	x 79	x 5,000
40	x 0.5	x 0.5	x 9	x 59	x 3,200
100	x ∼0.01	x ∼0.01	x 2	x 40	x 3,000

**FIGURE 15:**
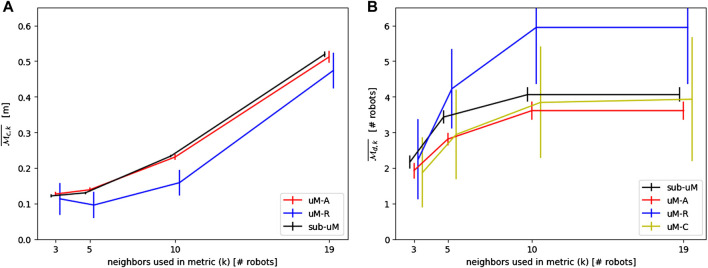
**(A)** Sensitivity analysis on the number of neighbors used in the continuous metric $\bar{M_{c,k}}$ . **(B)** Sensitivity analysis on the number of neighbors used in the continuous metric $\bar{M_{d,k}}$ .

A potential limitation of this work is the choice of using precisely three robots for both $\bar{M_{c}}$ and $\bar{M_{d}}$ to obtain comparable metrics for different flock sizes. Therefore, using a flock size of 20 robots, a sensitivity analysis has been performed using *k* = [3, 5, 10, 19] neighbors for the calculations of $\bar{M_{c,k}}$ and $\bar{M_{d,k}}$ respectively. The resulting performance of all models is reported in Figures 15A,B. No significant impact on the accuracy of the modeling can be observed, with the exception of *μ*M-R. This decrease in performance can be explained by the fact that *μ*M-R has been designed to model the *local* neighborhood of the central robot. However, taking more robots into account for the metric results in gradually taking into account the whole flock. Consequently, a local neighborhood representation is not as adequate as for smaller *k*’s.

An additional, related limitation is the extrapolation from the case study used here to “generality”. Even though the stochasticity of the flocking in this work is already severely limited, it is larger than for other spatially coordinated movement, such as formation control using model predictive control. While we believe the calibration techniques used in this work to adapt *μ*M-R and *μ*M-C to the limited amount of stochasticity will still be valid for more extreme cases, this still needs to be validated in future works. However, for collective movements with a similar (or higher) amount of stochasticity, the results obtained here remain valid as long as the other requirements of the respective models are fulfilled, as discussed below.

In this paper, we focused our efforts on pure modeling. Naturally, depending on the final purpose of the modeling effort, the metrics $\bar{M}$ need to be adapted accordingly. However, the two metrics $\bar{M_{c}}$ and $\bar{M_{d}}$ used here allow us to demonstrate several characteristics of microscopic modeling.

• While *μ*M-C always needs a discrete space, the corresponding discretization can be chosen arbitrarily. As a result, *μ*M-C can be applied to any case study, as long as the metric (and thus the model space) can be discretized.
• The structure of a model is easier to choose than the parameters. This is apparent in the fact that we could easily ground the structure of our *μ*M-C on our understanding of flocking and geometrical reasoning. However, to calibrate the parameters, dedicated experiments were necessary. Similarly, the structure of *μ*M-R using recorded GMMs is rather straightforward, whereas the use of geometrical reasoning to avoid dedicated experiments is strongly dependent on in-depth knowledge of the flocking behavior and the control parameters.
• The more stochastic interactions are present in the scenario, the more the well-mixed assumption is fulfilled, and the less calibration due to spatiality is necessary to create a quantitatively correct probabilistic microscopic model (*μ*M-C). While we are unable to prove this statement through this work alone, it becomes apparent by comparing with previous works leveraging the same type of microscopic models. In this work, the absence of stochastic interactions is apparent in our flocking implementation, as well as the resulting need for fine calibration, given the variability of the parameters recorded in Table 1. A similar situation can be observed in Correll and Martinoli (2006). However, as shown, for example, in Winfield et al. (2008), for a scenario similar to the one used here, though generating swarming rather than flocking, it has been possible to better estimate the model parameters through pure geometrical reasoning and therefore avoiding further fine-tuning through system identification techniques.
• The choice between *μ*M-A, *μ*M-R and *μ*M-C is a trade-off between the computational speed (*μ*M-C is the fastest), the modeling effort (*μ*M-A requires the least modeling choices), the modeling capability (*μ*M-C requires discretization), the modeling accuracy (*μ*M-A is most accurate) and the modeling precision (*μ*M-R has the highest standard deviation). Consequently, we are unable to name a generally valid “best microscopic modeling technique”. Nevertheless, we can give the following tentative recommendations as long as metrics are not too closely related to hardware.
• If a system including the metric of interest can be discretized, *μ*M-C provides the best modeling speedup with a limited number of calibration experiments needed, depending on the amount of stochasticity present.
• If the metric of interest cannot be discretized, *μ*M-C cannot be applied. Thus, *μ*M-R provides the best speedup possible without the need for calibration experiments, if the underlying behavior is known well enough for geometrical reasoning.
• If the modeling effort should be minimized, *μ*M-A provides a significant speedup over submicroscopic models, without the need for any important modeling or calibration effort.
• Submicroscopic models, *μ*M-A and *μ*M-R are all compatible with (high-level) robot control algorithms as used on real robots. *μ*M-C, on the other hand, requires the control algorithm to be discretized in order to construct the model. However, it should be noted that optimizing some key algorithmic parameters using *μ*M-C is still possible by explicitly exposing them in the discretized algorithm.
• With increasing abstraction, the amount of additional information that can be extracted from the model is reduced. From a sub-*μ*M, any desirable characteristics can be extracted as long as they are simulated (e.g., the temperature of batteries is usually not considered and could thus not be extracted). In *μ*M-A and *μ*M-R, the characteristics of the control input and the trajectory (e.g., speeds, accelerations, etc.) can be extracted. In *μ*M-C such additional information are not available, due to its non-spatial nature.
• Notably *μ*M-C, but to a certain degree also *μ*M-R, is composable. That is, if the overall behavior consists of sub-behaviors, for which the corresponding model exists, the overall model can be composed from the models of the sub-behaviors.
• Both *μ*M-C and *μ*M-R require strictly converging metrics for their respective parameter calibration. Consequently, oscillating or dynamic behavior metrics need to be cast in such a converging metric, for example by using the error of the resulting dynamic behavior to a behavior of reference. However, the use of *μ*M-C for such dynamic metrics is limited, as outlined previously.

While this work focused on the microscopic modeling level, the multi-level modeling framework in Martinoli et al. (2004) defines an additional level of modeling: the macroscopic one. Given the linearity of the *μ*M-C model in our scenario, a macroscopic version of it can be derived easily. However, the macroscopic model for *μ*M-R is not as straight forward, even for the considered scenario. In future work, we will therefore not only aim to demonstrate the applicability of *μ*M-R to different case studies but also expand it with a macroscopic model.

## 7 Conclusion

In this work, we have investigated the modeling of a spatially coordinated multi-robot system. More specifically, we concerned ourselves with a flocking scenario leveraging a potential field control law. We demonstrated the application of three different microscopic models for this case study. Using a fully spatial microscopic model (*μ*M-A), a very intuitive and widely used microscopic modeling type, we showed the impact of modeling precise kinematics. While even with precise kinematics, *μ*M-A did not match perfectly the underlying submicroscopic model considered as ground-truth, its modeling accuracy was considerable. We then demonstrated how to discretize spatiality to correctly capture the flocking scenario using a more canonical probabilistic microscopic model (*μ*M-C), resulting in a significantly increased speedup. We further proposed a novel microscopic model (*μ*M-R), situated between *μ*M-A and *μ*M-C in terms of both abstraction and simulation speedup, which is capable of accurately modeling the selected continuous flocking metric and reproduce any arbitrary control law deployable in lower level implementations, similarly to *μ*M-A. In an attempt to increase the usability of both *μ*M-R and *μ*M-C, we then showed how to leverage geometrical reasoning and system identification to avoid (for *μ*M-R) or reduce (for *μ*M-C) the need of prior experiments for the calibration of model parameters.

This contribution aims at transmitting our understanding of the different modeling techniques and demonstrating how they can be applied to spatially coordinated groups of robots. In a subsequent work, we plan to both extend the modeling techniques presented here to include macroscopic abstractions, and to apply them in order to optimize the underlying robot controllers.

## Data Availability

The raw data supporting the conclusions of this article will be made available by the authors, without undue reservation.
